# Enhanced photovoltaic properties of perovskite solar cells by TiO_2_ homogeneous hybrid structure

**DOI:** 10.1098/rsos.170942

**Published:** 2017-10-25

**Authors:** Pengyu Su, Wuyou Fu, Huizhen Yao, Li Liu, Dong Ding, Fei Feng, Shuang Feng, Yebin Xue, Xizhe Liu, Haibin Yang

**Affiliations:** 1 State Key Laboratory of Superhard Materials, Jilin University, Qianjin Street 2699, Changchun 130012, People's Republic of China; 2 Jilin Provincial Key Laboratory of Applied Atomic and Molecular Spectroscopy, Institute of Atomic and Molecular Physics, Jilin University, Changchun 130012, People's Republic of China

**Keywords:** TiO_2_ homogeneous hybrid structure, CH_3_NH_3_PbI_3_, perovskite solar cells

## Abstract

In this paper, we fabricated a TiO_2_ homogeneous hybrid structure for application in perovskite solar cells (PSCs) under ambient conditions. Under the standard air mass 1.5 global (AM 1.5G) illumination, PSCs based on homogeneous hybrid structure present a maximum power conversion efficiency of 5.39% which is higher than that of pure TiO_2_ nanosheets. The enhanced properties can be explained by the better contact of TiO_2_ nanosheets/nanoparticles with CH_3_NH_3_PbI_3_ and fewer pinholes in electron transport materials. The advent of such unique structure opens up new avenues for the future development of high-efficiency photovoltaic cells.

## 1. Introduction

TiO_2_ is an optimized candidate for the development of nanoscale architectures due to its appropriate electronic band structure, photostability, non-toxicity and high chemical inertness [1–9]. Among diverse TiO_2_ nanostructure morphologies, TiO_2_ nanosheets (TiO_2_NSs) have been widely applied in the field of photovoltaic cells due to their sufficient surface area [10]. Many endeavours have been made to fabricate perovskite solar cells (PSCs) based on TiO_2_NSs films [11,12]. Although such PSCs based on TiO_2_NSs films show promising photovoltaic performance, poor properties are shown after the contact of CH_3_NH_3_PbI_3_ (MAPbI_3_) and fluorine-doped tin oxide (FTO) along the pinholes in the TiO_2_NSs films. Given all that, microscopic and high specific surface area TiO_2_ nanoparticles (TiO_2_NPs) can be introduced to wipe out the pinholes and retain high specific surface area of TiO_2_NSs [13–16]. Once TiO_2_NPs were deposited onto TiO_2_NSs, a peculiar TiO_2_NSs/NPs homogeneous hybrid structure can be formed. This structure is anticipated to simultaneously possess higher specific surface area and fewer pinholes. Hence, it will definitely increase the power conversion efficiency of PSCs. However, up to now, scarce relevant works have been reported on those issues.

Here, we report a TiO_2_NSs/NPs homogeneous hybrid structure as electron transport material (ETM) in PSCs, which was fabricated by the simple hydrothermal and chemical bath deposition (CBD) method. The photovoltaic properties of PSCs based on TiO_2_NSs/NPs homogeneous hybrid structure are superior to those based on bare TiO_2_NSs due to the introduction of TiO_2_NPs. This homogeneous hybrid structure may provide a new strategy for improving the performance of PSCs.

## 2. Experimental procedure

### 2.1. Experimental

The compact TiO_2_ (c-TiO_2_) and TiO_2_NSs films were synthesized by the simple CBD and hydrothermal method [17,18]. TiO_2_NSs films were fabricated by a hydrothermal method at 170°C for 3 h and annealed at 550°C for 2 h in air. To deposit TiO_2_NPs, the FTO/c-TiO_2_/TiO_2_NSs substrates were placed into an aqueous solution with 0.07 M TiCl_4_ at 70°C for 30 min. In addition, the TiO_2_NPs films were annealed at 450°C for 15 min in air atmosphere. The entire procedure of fabricating TiO_2_NPs films was termed as one cycle (C). Here, the procedure was repeated for 1C, 3C, 5C, 7C and 9C. Finally, the samples were annealed at 450°C for 30 min under ambient conditions and taken out after cooling down to room temperature.

The TiO_2_ substrates were treated by UV–ozone for 15 min before the MAPbI_3_ was deposited. Then, 50 µl PbI_2_ (462 mg ml^−1^ in dimethylformamide) was spin-coated onto the substrates (1.5 cm ×1.5 cm) with a low speed of 500 r.p.m. for 5 s and with a high speed of 4000 r.p.m. for 30 s. After that, the substrates were annealed at 100°C for 20 min. CH_3_NH_3_I (MAI) was synthesized by the technique as described in [19]. In the next step, 200 µl MAI (10 mg ml^−1^ in isopropanol) was spin-coated onto the substrates. After rinsing with isopropanol, the perovskite films were annealed at 100°C for 40 min. Thirty millilitre hole transport material (HTM) as described in [19] was spin-coated onto the perovskite films at 4000 r.p.m. for 10 s (0.073 g 2,2′,7,7′-tetrakis(*N*,*N*-di-*p*-methoxyphenylamine)-9,9′-spirobifluorene, 29 µl of 4-tert-butylpyridine, 18 µl of a lithium-bis(trifluoromethanesulfonyl)imide (Li-TFSI) solution (520 mg Li-TFSI/1 ml acetonitrile) were dissolved in 1 ml chlorobenzene) [20]. Finally, the Ag back electrode was deposited by thermal evaporation.

### 2.2. Characterization

The microstructure and morphology of the films were observed by a MAGELLAN 400 scanning electron microscope. Transmission electron microscopy (TEM) and high-resolution TEM (HRTEM) measurements were conducted by a JEOL JEM-2100F microscope. The analysis of composition and crystal structure was conducted using X-ray diffraction (XRD; Rigaku D/max-2500) using Cu Kα radiation (*λ* = 1.5418 Å). Ultraviolet–visible (UV-vis) absorption spectra were measured in the range from 200 to 1000 nm by a UV-3150 double-beam spectrophotometer at room temperature. The current–voltage curves of PSCs were recorded by a Keithley 2400 source measure unit.

## 3. Results and discussion

### 3.1. Scanning electron microscope images

**Figure 1.**
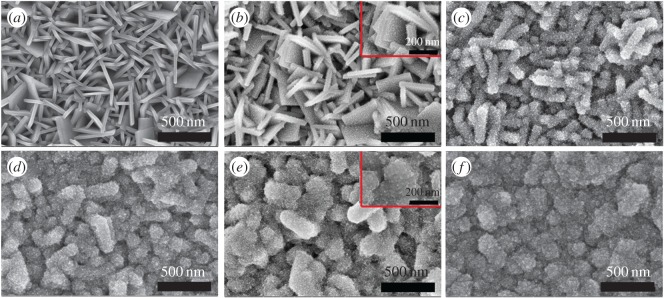
Top-view SEM images of TiO_2_NPs deposited on TiO_2_NSs for (*a*) 0C, (*b*) 1C, (*c*) 3C, (*d*) 5C, (*e*) 7C and (*f*) 9C. The insets in (*b*) and (*e*) represent the enlarged images of TiO_2_NSs/NPs of 1C and 7C, respectively.

In figure 1, we present top-view scanning electron microscopy (SEM) images of the TiO_2_NSs array films after being combined with TiO_2_NPs for different cycles. TiO_2_NSs are coated with TiO_2_NPs uniformly, and the surface of the TiO_2_NSs becomes rough gradually. There was a notable increase in the adsorption of TiO_2_NPs deposited onto the TiO_2_NSs films, and the TiO_2_NPs architecture could result in a full TiO_2_NSs/NPs homogeneous hybrid film. The inset of figure 1*b* represents an enlarged image of TiO_2_NSs deposited with TiO_2_NPs of 1C. Because of the high surface energy and reactivity of {001} facets, the TiO_2_NSs can adsorb more particles on this facet. The inset of figure 1*e* represents an enlarged view of TiO_2_NPs deposited on TiO_2_NSs for 7C.

**Figure 2.**
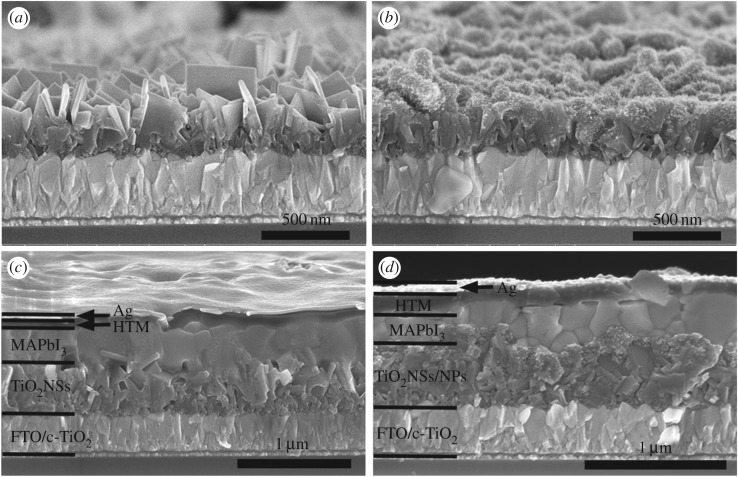
Cross-sectional SEM images of (*a*) TiO_2_NSs films grown on FTO at 170°C for 3 h, (*b*) TiO_2_NSs after coated with 7CNPs, (*c*) device based on TiO_2_NSs and (*d*) device based on TiO_2_NSs/7CNPs.

As portrayed in figure 2*a*, the aligned TiO_2_NSs films are relatively smooth and vertically grew on the FTO/c-TiO_2_ substrates, providing the transfer path for electrons. The thickness of the film is approximately 370 nm. Figure 2*b* is the SEM image of TiO_2_NSs film after the deposition of 7C TiO_2_NPs; obviously, the surface of the TiO_2_NSs became rougher, which benefits for the combination of MAPbI_3_ [21]. The cross-sectional SEM images of PSC devices with and without TiO_2_NPs are presented in figure 2*c*,*d*, respectively. MAPbI_3_ crystals are compactly arranged on the surface of TiO_2_ substrates. When the ETM films are not coated with TiO_2_NPs, some pinholes are seen, as shown in figure 2*c*. However, no pinholes are seen in the TiO_2_NSs/NPs/MAPbI_3_ films which could be ascribed to the full TiO_2_NSs/NPs film, and higher power conversion efficiency (PCE) was obtained.

### 3.2. Transmission electron microscopy and high-resolution transmission electron microscopy observation

**Figure 3.**
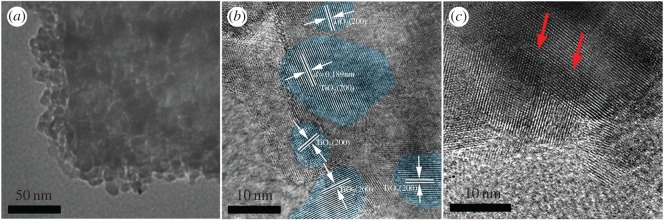
(*a*) TEM bright-field image of TiO_2_NSs/NPs and (*b*,*c*) HRTEM images of TiO_2_NSs/NPs.

The detailed microscopic characterization of TiO_2_NSs/NPs nanostructures was performed by TEM, as shown in figure 3. Figure 3*a* clearly displays that TiO_2_NPs uniformly grew on TiO_2_NSs. This homogeneous structure was not damaged through ultrasonic processing, signifying that the TiO_2_NSs and TiO_2_NPs bonded firmly together. To clarify the growth mechanism, HRTEM was carried out. The inter-planar distance between the neighbouring lattice fringes of HRTEM is 0.189 nm which is corresponding to the inter-planar distance from {200} planes of TiO_2_NPs (JCPDS no. 21–1272), as shown in figure 3*b*. The TEM and HRTEM images indicate that TiO_2_NPs exhibit homogeneous fine grain microstructure, with an average grain size of approximately 10–20 nm. The staggered lattice of TiO_2_NSs and TiO_2_NPs was also observed by HRTEM and the joint parts are pointed out by the arrows in figure 3*c*. The plentiful growth interfaces inevitably will result in more charge transfer channels, which can enhance the electronic injection efficiency.

### 3.3. Phase composition and phase structure

**Figure 4.**
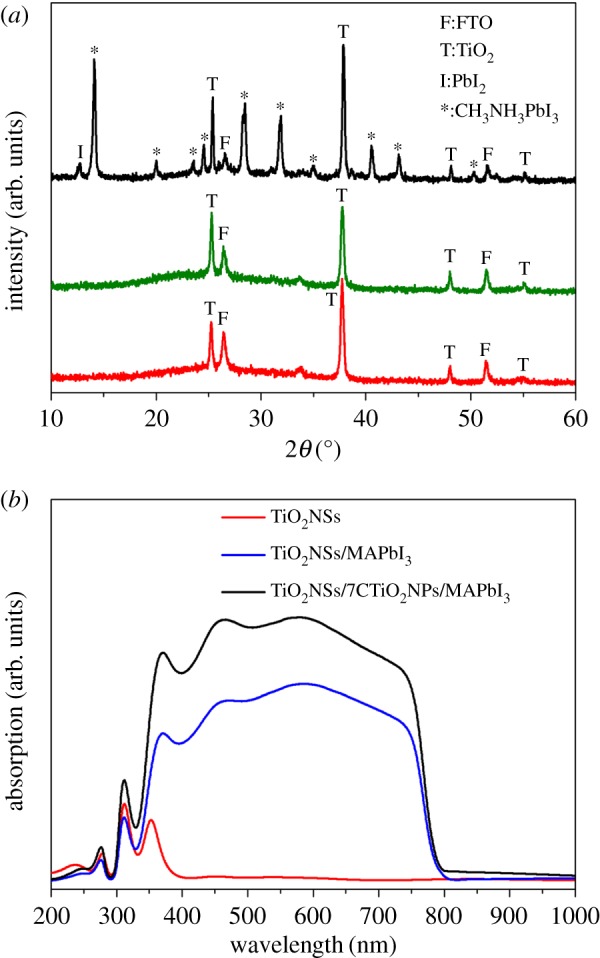
(*a*) X-ray diffraction patterns of (from bottom to top) FTO/c-TiO_2_/TiO_2_NSs, FTO/c-TiO_2_/TiO_2_NSs/7CNPs and FTO/c-TiO_2_/TiO_2_NSs/7CNPs/MAPbI_3_. The plot shows the X-ray intensity as a function of 2*θ*. (*b*) UV-vis absorption spectra of films.

The prepared TiO_2_ and TiO_2_/MAPbI_3_ films were examined by XRD to characterize the crystal structure (figure 4*a*). The diffraction pattern of TiO_2_NSs array film matches fairly well with the existing reference data available for this crystal from the Joint Committee on Powder Diffraction Standards (JCPDS no. 21–1272). In addition, after depositing with TiO_2_NPs for 7C, no diffraction lines of impurities were detected. The black curve shows the XRD pattern of FTO/c-TiO_2_/TiO_2_NSs/NPs/MAPbI_3_ film. Except the diffraction peaks of FTO, TiO_2_ and a weak peak at 12.7°of PbI_2_, other diffraction peaks all correspond to MAPbI_3_. This indicates that the MAPbI_3_ has a high purity and has been successfully coated onto the surface of TiO_2_ films. The peaks of MAPbI_3_ were sharp, indicating that MAPbI_3_ possesses good crystallinity.

### 3.4. Ultraviolet–visible absorption spectroscopy

The UV-vis absorption spectra of bare TiO_2_NSs array films, TiO_2_NSs/MAPbI_3_ films and TiO_2_NSs/NPs/MAPbI_3_ films are plotted in figure 4*b* as a function of wavelength. The peak maximum of TiO_2_NSs films occurs at approximately 370 nm and the films have no significant absorption for visible light due to the large band gap of TiO_2_ [22,23]. However, after coating with MAPbI_3_, the TiO_2_NSs/MAPbI_3_ films present excellent absorption capacity from 350 to 800 nm, and the absorption intensity increased substantially. The variation indicates that the deposited MAPbI_3_ has significantly extended the photoresponse of TiO_2_NSs films to the visible light region. The most striking thing is that after TiO_2_NSs films combined with TiO_2_NPs for 7 C, the TiO_2_NSs/NPs/MAPbI_3_ films exhibit stronger absorption than TiO_2_NSs/MAPbI_3_ films. This result indicates that the MAPbI_3_ films combine with TiO_2_NSs/NPs films better than TiO_2_NSs films.

### 3.5. Photovoltaic characterization of perovskite solar cells based on TiO_2_NSs/NPs films

**Figure 5.**
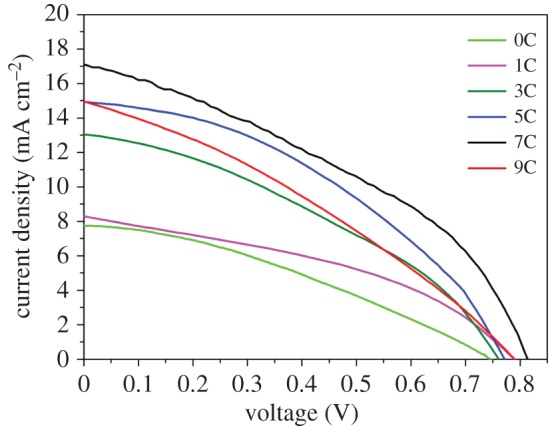
*J–V* characteristic of the lead iodide perovskite solar cells based on TiO_2_NSs/NPs films of different deposition cycles.

To probe the effect of different amount of TiO_2_NPs on photovoltaic performance, successive deposition cycle (C) experiments of TiO_2_NPs were conducted. The working area of devices was 0.15 mm^2^. All current–voltage curves were recorded in air and derived from reverse scan (the bias scan rate was 100 mV s^−1^). Figure 5 shows the *J–V* characteristics of the PSCs under one sun AM 1.5G irradiance, and the corresponding parameters are summarized in table 1. The *J–V* characteristics are enhanced with the increase of TiO_2_NPs in the early cycles, suggesting that a higher incorporated amount of TiO_2_NPs induced more superior photovoltaic characteristics. Surprisingly, as the amount of TiO_2_NPs increased upon the cycles to seven, the PSCs exhibited a best PCE of 5.39%, with *V*_oc_ = 0.82 V, *J*_sc_ = 17.06 mA cm^−2^ and FF = 0.40.

**Table 1.** Photovoltaic device parameters of the TiO_2_NSs/NPs/CH_3_NH_3_PbI_3_ solar cells.

cycles	*J*_sc_ (mA cm^−2^)	*V*_oc_ (V)	FF	PCE
0	4.27	0.68	0.30	0.86
1	6.59	0.8	0.38	2.07
3	12.91	0.78	0.37	3.72
5	14.65	0.79	0.42	4.8
7	17.06	0.82	0.40	5.39
9	14.87	0.79	0.35	3.89

In the early 7 cycles, the *V*_oc_ significantly increased along with the decreased pinholes which will generate charge loss. After TiO_2_NPs are deposited onto TiO_2_NSs, the MAPbI_3_ grain size tends to be larger than that of pure TiO_2_NSs. Larger grain size leads to fewer grain boundaries and, consequently, the corresponding *J*_sc_ improved [24]. With the increase of TiO_2_NPs, the surface of TiO_2_NSs array films appears rougher. This rougher surface is favourable for the combination of MAPbI_3_ and it will improve the fill factor of PSCs. However, when the deposited cycles are more than seven, the device performance of PSCs dropped. This phenomenon is presumably attributed to the excessive TiO_2_NPs grain boundaries. When TiO_2_NSs/NPs films transported photo-induced electrons from MAPbI_3_ to FTO, more grain boundaries caused more electron annihilation. This inevitably will decrease the *J–V* characteristics of PSCs. Etgar *et al*. [11] fabricated MAPbI_3_/TiO_2_NSs solar cell with *J*_sc_ = 16.1 mA cm^−2^, *V*_oc_ = 0.631 V, corresponding to a PCE of 5.5% under glovebox conditions. Compared with their PSCs based on pure TiO_2_NSs, the TiO_2_ homogeneous hybrid structure PSCs synthesized under air conditions exhibit higher *J*_sc_, *V*_oc_ and compatible PCE.

**Scheme 1.**
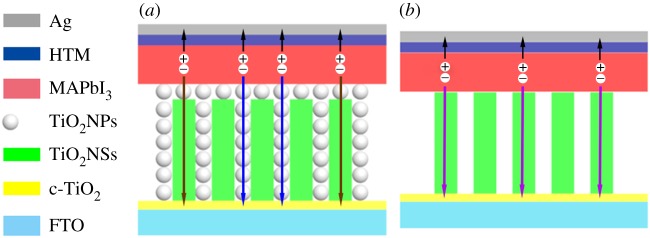
Schematic of PSCs based on (*a*) TiO_2_NSs/NPs homogeneous hybrid structure films and (*b*) pure TiO_2_NSs films.

Holes are transported to the counter electrode by HTM in scheme 1*a* (the short black arrows). Electrons are transported to FTO by TiO_2_NPs (the long blue arrows) and TiO_2_NSs/NPs (the long brown arrows). Compared with pure TiO_2_NSs films, fewer pinholes exist in TiO_2_NSs/NPs homogeneous hybrid films, and these hybrid films have higher specific surface area. Therefore, the photovoltaic performance of PSCs based on TiO_2_NPs/NSs homogeneous hybrid structure is significantly enhanced.

## 4. Conclusion

We fabricated a TiO_2_NSs/NPs homogeneous hybrid structure, the ETM in PSCs, by the hydrothermal and CBD method. When TiO_2_NPs are deposited onto TiO_2_NSs for 7 cycles, the PSCs based on TiO_2_NPs/NSs homogeneous hybrid structure exhibit photovoltaic performance with a best efficiency of 5.39% under one sun AM 1.5G solar spectrum, which is two and a half times that of bare TiO_2_NSs. Compared with pure TiO_2_NSs films, the MAPbI_3_ combined better with rough and free of pinholes TiO_2_NSs/NPs homogeneous hybrid films. Hence, it demonstrates that this extraordinary structure will have potential application for enhancing photovoltaic performance of PSCs in the future.

## Supplementary Material

Preparation of c-TiO_2_ films; Preparation of TiO_2_NSs films; SEM Images; J-V characteristic; Tables

## Data accessibility

The datasets supporting this article have been uploaded as part of the electronic supplementary material.
