# Supplementary material for: Enhanced photovoltaic properties of perovskite solar cells by TiO2 homogeneous hybrid structure
Source: R Soc Open Sci. 2017 Oct 25;4(10):170942. doi: 10.1098/rsos.170942 (PMC5666275; doi:10.1098/rsos.170942)
Supplement: Preparation of c-TiO2 films; Preparation of TiO2NSs films; SEM Images; J-V characteristic; Tables [file rsos170942supp1.doc]

**Royal Society Open Science**

**Supplementary Material**

**Enhanced Photovoltaic Properties of Perovskite Solar Cells by TiO_2_ Homogeneous Hybrid Structure**

Pengyu Su^1^, Wuyou Fu^1^, Huizhen Yao^1^, Li Liu^1^, Dong Ding^1^, Fei Feng^1^, Shuang Feng^1^, Yebin Xue^2^, Xizhe Liu^2^, Haibin Yang^1^

^1^ *State Key Laboratory of Superhard Materials, Jilin University, Qianjin Street 2699,Changchun,*

*130012, People’s Republic of China*

^2^ Jilin Provincial Key Laboratory of Applied Atomic and Molecular Spectroscopy, Institute of Atomic and Molecular Physics, Jilin University, Changchun, 130012, China

**Author for correspondence:** Wuyou Fu

E-mail: [fuwy@ jlu.edu.cn](mailto:fuwy@jlu.edu.cn)

**Preparation of c-TiO_2_ films:** At first, FTO glass substrates were cleaned by ultrasonication for 20 min with deionized water, acetone, isopropanol and ethyl alcohol successively. After blow-dried by N_2_ flow, the c-TiO_2_ films were deposited onto the clean FTO by chemical bath deposition (CBD) method with 0.06 M TiCl_4_ aqueous solution at 70 ℃ for 30 min. The c-TiO_2_ films were annealed at 450 ℃ for 30 min in ambient conditions.

**Preparation of TiO_2_NSs films:** The TiO_2_ nanosheets films were fabricated as follows. The precursor solution was prepared by mixed 15 mL of deionized water and 15 mL of concentrated hydrochloric acid (mass fraction 36.5-38%). After stirred for 5 min, 0.5 mL of titanium butoxide (TBT) was added into the mixed solution. The solution was stirred for 20 min before 0.25 g ammonium hexafluorotitanate ((NH_4_)_2_TiF_6_) was added into it. After that, the solution was stirred for 5 min. Then, a FTO/c-TiO_2_ substrate was put into a Teflon-lined steel autoclave with volume of 50 mL. The solution was poured into the autoclave and it was placed into an electric oven at 170 ℃ for 1 h, 2 h, 3 h and 4 h. After cooling to room temperature, the substrates were taken out and washed with distilled water. Finally, the TiO_2_NSs films were annealed at 550 ℃ for 1 h in the air.

**
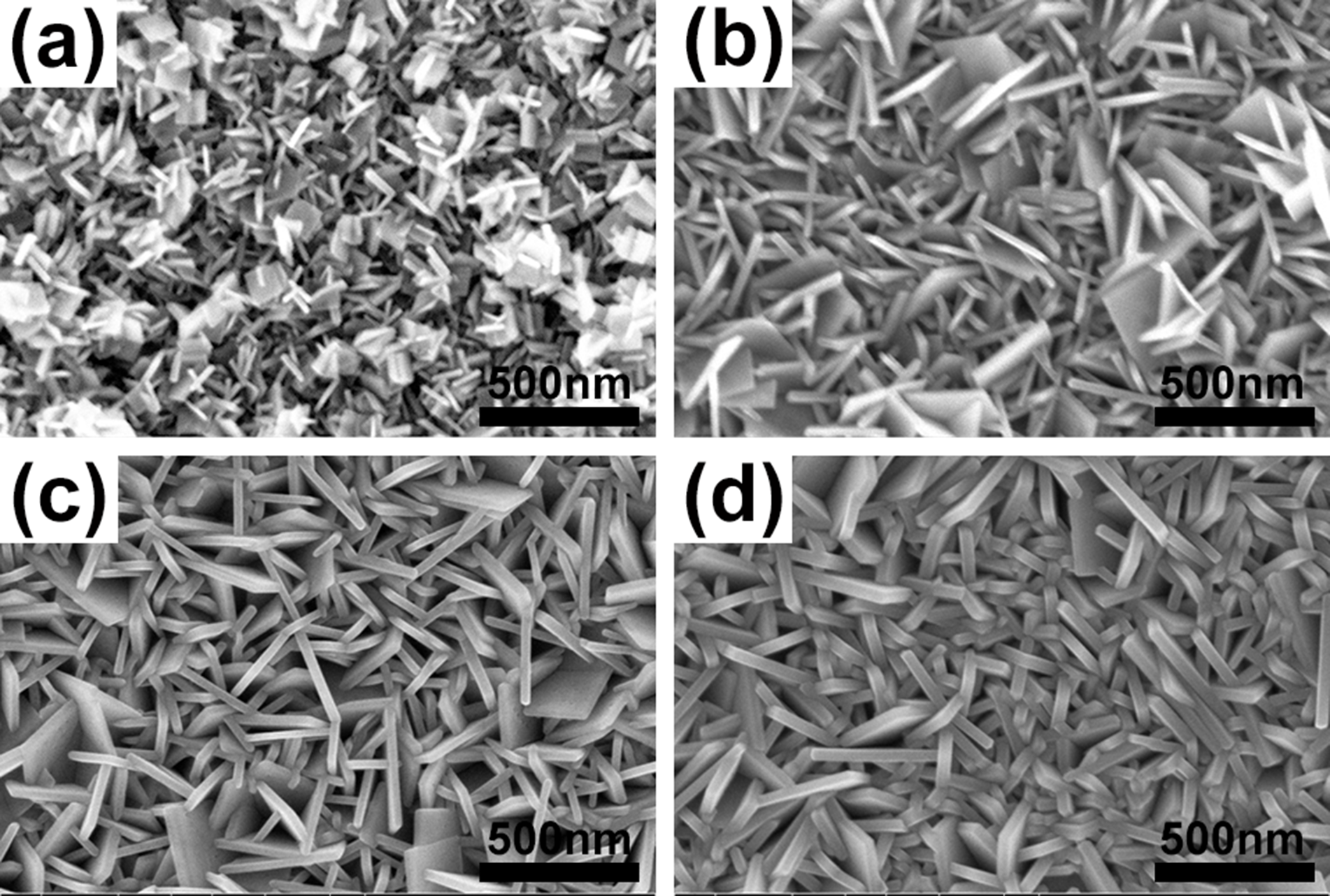
**

**Fig. S1** Top-view images of TiO_2_NSs on FTO/c-TiO_2_ prepared at 170 ℃ for (a) 1 h; (b) 2 h; (c) 3 h; (d) 4 h, the scale bar is 500nm.

To obtain an optimal candidate which is suitable to fabricate the ETM for PSCs, we altered the reaction time to obtain TiO_2_NSs arrays with different size and density. The shape of the TiO_2_NSs changes obviously (Fig. S1). As shown in Fig. S1a, it is clear that the TiO_2_NSs grow on the FTO vertically in the early stage. Then, the TiO_2_NSs clusters are formed on the vertical TiO_2_NSs. The side length of a single TiO_2_NSs is 90 nm, and with a thickness of 5 nm. When the reaction time increased to 2 h, the TiO_2_NSs become bigger, with a side length of 200 nm and the thickness of 25 nm. Interestingly, no clusters form on the TiO_2_NSs films (Fig. S1b). In Fig. S1c, as the reaction time is prolonged, the TiO_2_NSs arrays are more orderly than in 2 h. The side length of TiO_2_NSs is 370 nm and the thickness is 30 nm. Fig. S1d presents the TiO_2_NSs films of 4 h and one can see that the TiO_2_NSs become denser compared with 3 h. The thickness of TiO_2_NSs increases to 40 nm, and the side length has no change.


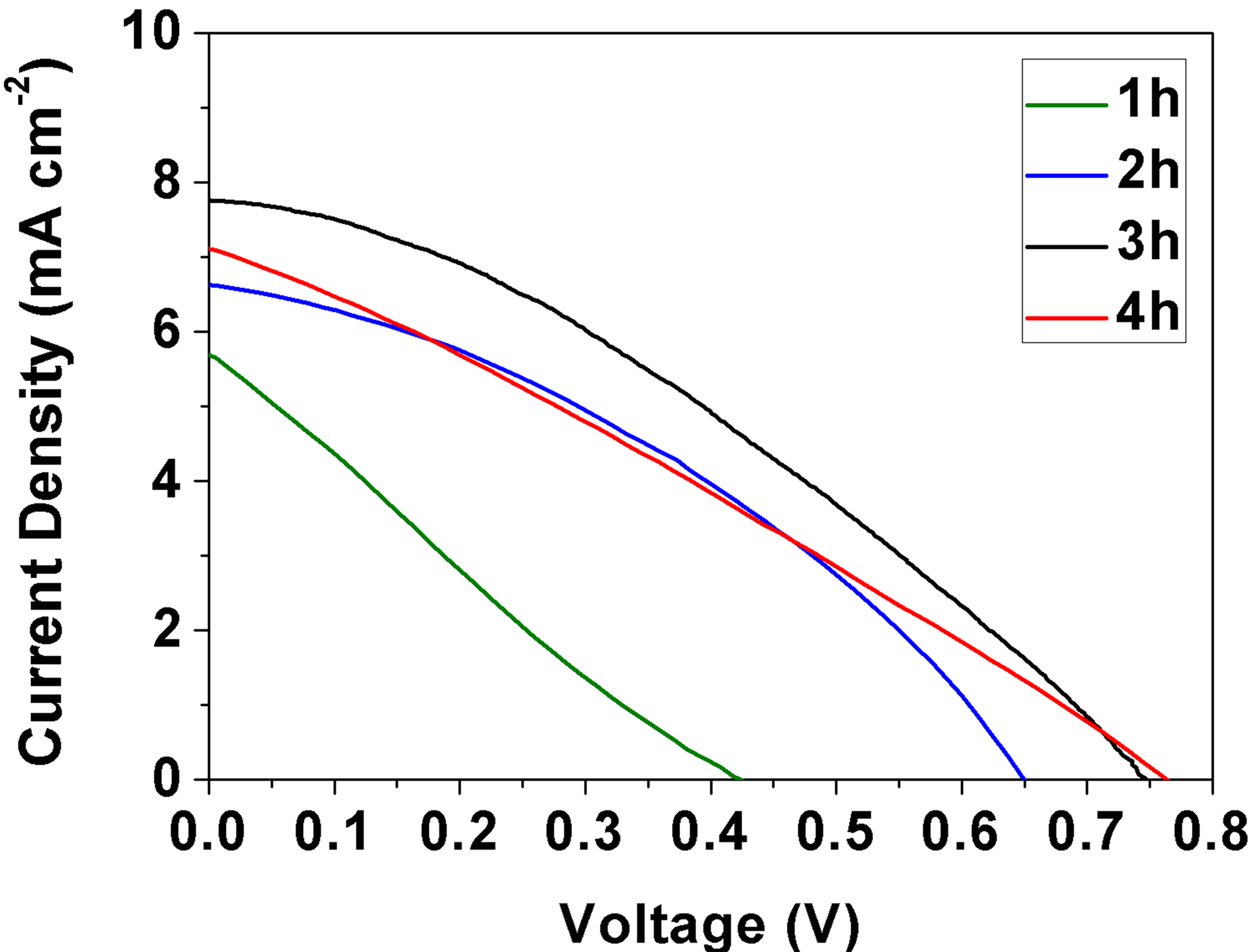


**Fig. S2** *J-V* characteristic of the lead iodide perovskite solar cells based on TiO_2_NSs films of different reaction time.

**Table. S1** Photovoltaic Device Parameters of the TiO_2_NSs/CH_3_NH_3_PbI_3_ Solar Cells.

| Reaction time | *J_sc_* (mA cm^-2^) | *V_oc_* (V) | FF | PCE (%) |
| --- | --- | --- | --- | --- |
| 1h | 5.70 | 0.42 | 0.23 | 0.56 |
| 2h | 6.66 | 0.65 | 0.36 | 1.77 |
| 3h | 7.76 | 0.75 | 0.35 | 2.05 |
| 4h | 7.06 | 0.76 | 0.30 | 1.58 |

Fig. S2 shows the *J-V* characteristics of TiO_2_NSs/MAPbI_3_ photovoltaic solar cells of different TiO_2_NSs reaction time under AM 1.5G condition. The PSCs show a low PCE when the [hydrothermal](http://cn.bing.com/dict/search?q=hydrothermal&FORM=BDVSP6&mkt=zh-cn) reaction time is 1 h. This problem can be explained by the thin TiO_2_NSs films which are hard to combine with the MAPbI_3_. This phenomenon was alleviated by increasing of the reaction time and the PCE of PSCs improved. As the reaction time reached to 3 h, the PSCs show the best photovoltaic performance with *V*_oc_ = 0.75 V, *J*_sc_ = 7.76 mA cm^-2^, FF = 0.35, corresponding to a PCE of 2.05% (Table S1). When the reaction time was prolonged to 4h, the PCE declined due to the high density and low specific surface area of TiO_2_NSs.

**
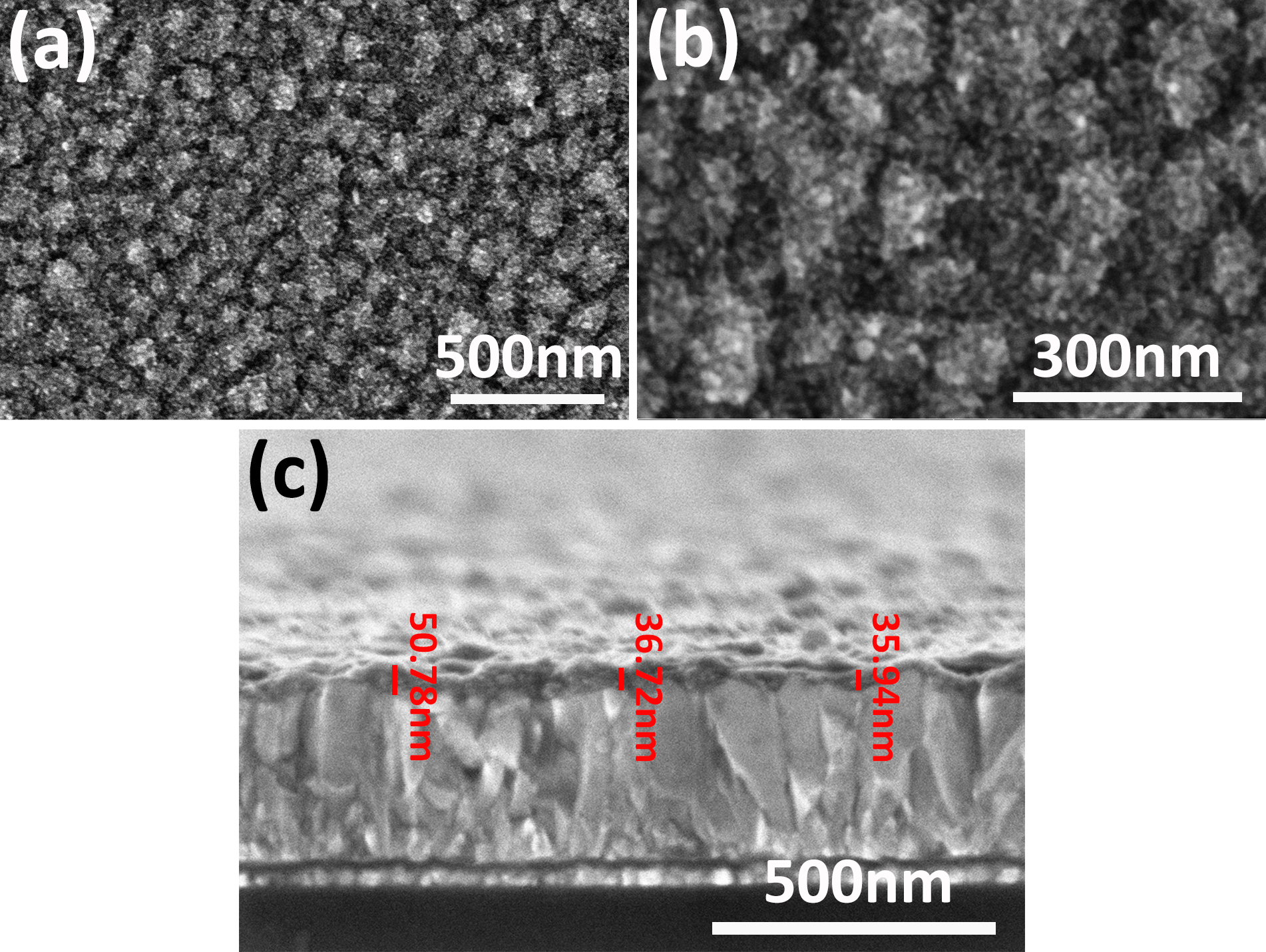
**

**Fig. S3** (a) Top-view image of 7C TiO_2_NPs on FTO/c-TiO_2_; (b) the enlarged view of TiO_2_NPs; (c) cross-sectional SEM image of 7C TiO_2_NPs on FTO/c-TiO_2_.


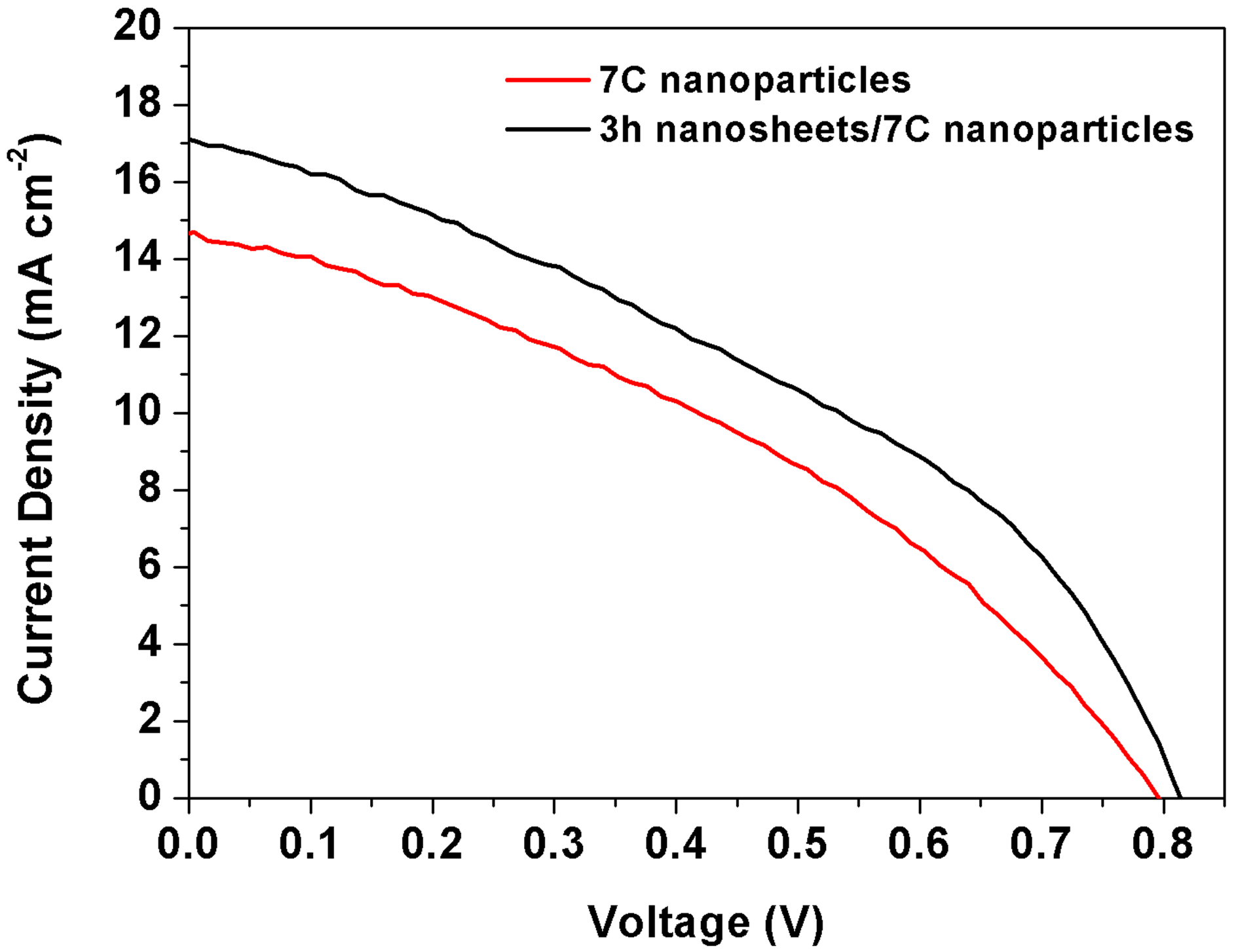


**Fig. S4** *J-V* characteristic of the lead iodide perovskite solar cells based on 3h TiO_2_NSs/7CNPs and 7C NPs films.

**Table. S2** Photovoltaic Device Parameters of the FTO/c-TiO_2_/TiO_2_NSs/NPs/CH_3_NH_3_PbI_3_/HTM/Ag and FTO/c-TiO_2_/TiO_2_NPs/CH_3_NH_3_PbI_3_/HTM/Ag Solar Cells.

| substrates | *J_sc_* (mA cm^-2^) | *V_oc_* (V) | FF | PCE (%) |
| --- | --- | --- | --- | --- |
| 7C NPs | 14.7 | 0.79 | 0.37 | 4.32 |
| 3h NSs/7C NPs | 17.06 | 0.82 | 0.40 | 5.39 |

As comparison, we fabricated PSCs based on 7C TiO_2_NPs, Fig. S3 is the SEM image of FTO/c-TiO_2_/7C TiO_2_NPs. Fig. S3a is the top-view image of TiO_2_NPs, and Fig. S3b is the enlarged view. As can be seen, TiO_2_NPs grew on FTO/c-TiO_2_ substrate uniformly. Fig. S3c is the cross-sectional SEM image of 7C TiO_2_NPs on FTO/c-TiO_2_, the thickness of TiO_2_NPs is 41.15nm. Fig. S4 shows the *J-V* characteristics of PSCs based on FTO/c-TiO_2_/7C TiO_2_NPs, and the corresponding parameters are summarized in Table S2. Under one sun AM 1.5G [irradia](http://cn.bing.com/dict/search?q=irradiation&FORM=BDVSP6&mkt=zh-cn)nce, PSCs based on FTO/c-TiO_2_/7C TiO_2_NPs exhibited a best PCE of 4.32%, with *V_oc_* = 0.79 V, *J_sc_* = 14.7 mA cm^-2^, and FF = 0.37. PSCs based on FTO/c-TiO_2_/3h NSs/7C TiO_2_NPs show surperior photovoltaic characterization.


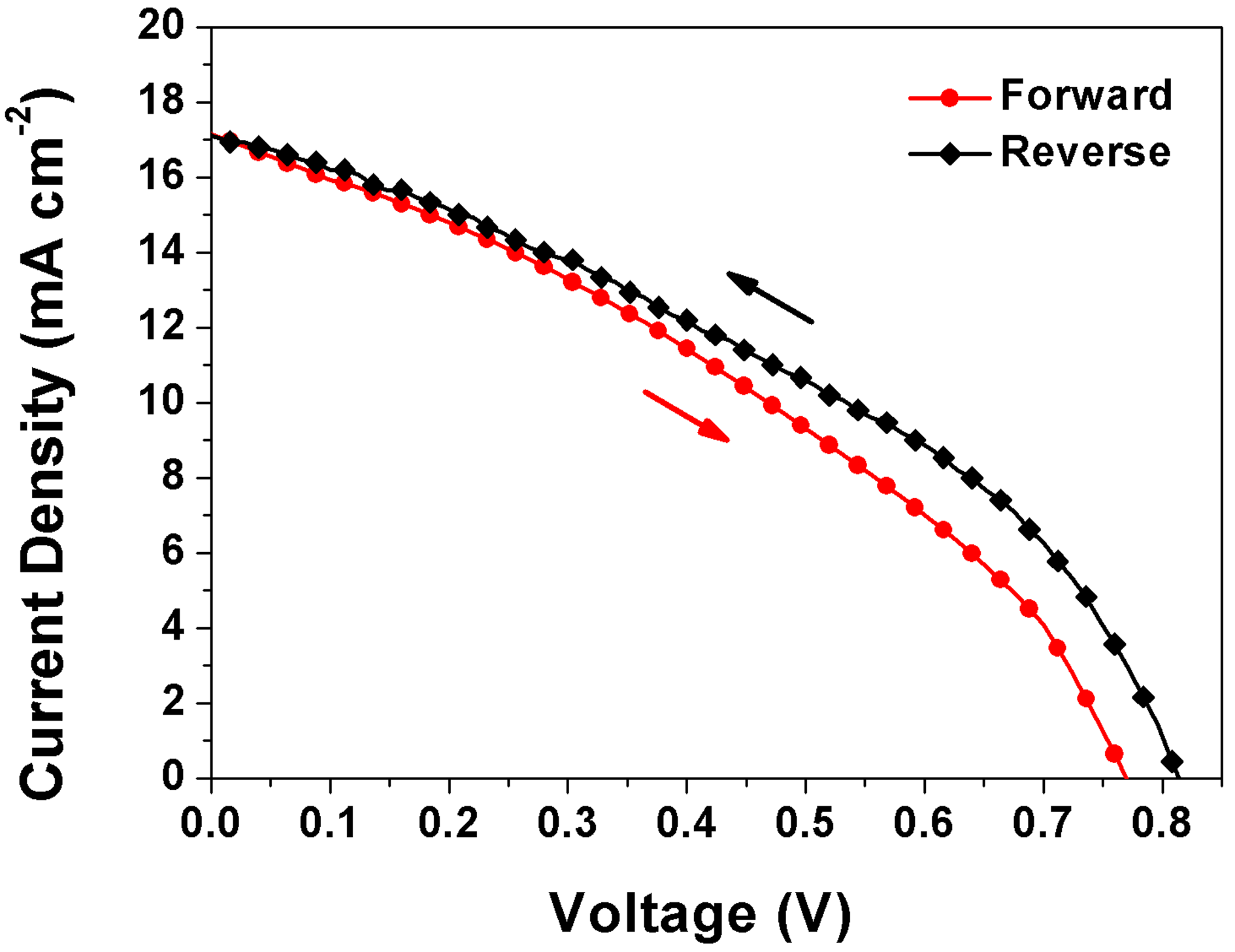


**Fig S5.** *J-V* curves of perovskite solar cell devices FTO/c-TiO_2_/TiO_2_NSs/7C NPs/CH_3_NH_3_PbI_3_/HTM/Ag: Red line (forward scan), Black line (reverse scan)

**Table. S3** Photovoltaic Device Parameters of the FTO/c-TiO_2_/TiO_2_NSs/7C NPs/CH_3_NH_3_PbI_3_/HTM/Ag.

|  | *J_sc_* (mA cm^-2^) | *V_oc_* (V) | FF | PCE (%) |
| --- | --- | --- | --- | --- |
| Forward | 17.18 | 0.77 | 0.35 | 4.68 |
| Reverse | 17.06 | 0.82 | 0.40 | 5.39 |
